# Single atom changes in newly synthesized HIV protease inhibitors reveal structural basis for extreme affinity, high genetic barrier, and adaptation to the HIV protease plasticity

**DOI:** 10.1038/s41598-020-65993-z

**Published:** 2020-06-30

**Authors:** Haydar Bulut, Shin-ichiro Hattori, Hiromi Aoki-Ogata, Hironori Hayashi, Debananda Das, Manabu Aoki, David A. Davis, Kalapala Venkateswara Rao, Prasanth R. Nyalapatla, Arun K. Ghosh, Hiroaki Mitsuya

**Affiliations:** 1 HIV and AIDS Malignancy Branch, National Cancer Institute, National Institutes of Health, Bethesda, 20892 MD United States; 2 Department of Refractory Viral Infections, National Center for Global Health and Medicine Research Institute, Tokyo, 162-8655 Japan; 3 Department of Intelligent Network for Infection Control, Tohoku University Graduate School of Medicine, 2-1, Seiryo-machi, Aoba-ku, 980-8575, Sendai, Miyagi, Japan; 4 Department of Chemistry and Department of Medicinal Chemistry and Molecular Pharmacology, Purdue University, West Lafayette, 47907 IN United States; 5 Department of Clinical Sciences, Kumamoto University Hospital, Kumamoto, 860-8556 Japan

**Keywords:** Chemical biology, Drug discovery, Structural biology

## Abstract

HIV-1 protease inhibitors (PIs), such as darunavir (DRV), are the key component of antiretroviral therapy. However, HIV-1 often acquires resistance to PIs. Here, seven novel PIs were synthesized, by introducing single atom changes such as an exchange of a sulfur to an oxygen, scission of a single bond in P2′-cyclopropylaminobenzothiazole (or -oxazole), and/or P1-benzene ring with fluorine scan of mono- or bis-fluorine atoms around DRV’s scaffold. X-ray structural analyses of the PIs complexed with wild-type Protease (PR_WT_) and highly-multi-PI-resistance-associated PR_DRV_^R^_P51_ revealed that the PIs better adapt to structural plasticity in PR with resistance-associated amino acid substitutions by formation of optimal sulfur bond and adaptation of cyclopropyl ring in the S2′-subsite. Furthermore, these PIs displayed increased cell permeability and extreme anti-HIV-1 potency compared to DRV. Our work provides the basis for developing novel PIs with high potency against PI-resistant HIV-1 variants with a high genetic barrier.

## Introduction

Over the last 3 decades, HIV-1 infection has remained a devastating disease with ~80 million people infected worldwide and nearly half of them have lost lives from the infection (https://www.avert.org/global-hiv-and-aids-statistics). However, the present combined antiretroviral therapy (cART) has been proven to potently suppress HIV-1 replication and to significantly prolong the survival of people with HIV-1 infection and AIDS. The inhibitors of HIV-1 protease (PR), an essential enzyme that cleaves gag and gag-pol polyproteins into mature functional proteins, are the key element of effective cART^1,2^. Blocking the activity of PR leaves virions in an immature state, which are unable to infect cells, thus leading to restoration of the immune system^3^. Currently, there are nine licensed HIV-1 protease inhibitors (PIs) with nanomolar ranges of inhibitory  potency against wild type virus; however, none of them are able to eradicate the virus in the body^4,5^. Moreover, drug-resistant HIV-1 variants often emerge during long-term therapy^6,7^, due to the error-prone virally-encoded reverse transcriptase (RT)^8^.

Darunavir (DRV) is a second-generation nonpeptidic PI, which is highly potent against wild-type HIV-1 (HIV_WT_) and has a high genetic barrier to the emergence of DRV-resistant variants, retaining its potent anti-HIV activity over long-term periods in clinical settings^9,10^. Nevertheless, DRV-resistant variants have been reported and there is urgent need to develop more potent and resistance-repellent PIs^4,11^. Based on the DRV scaffold, numerous attempts have been made to develop more potent inhibitors, which possibly adapt to the PR-binding pocket(s) deformed by amino acid substitutions^11^. Structure-based optimizations have mainly focused on increasing the number of hydrogen bond interactions and on effectively filling the hydrophobic sub-pockets S1/S2 to establish more favorable van der Waals (vdW) interactions^1,12–14^.

**Figure 1:**
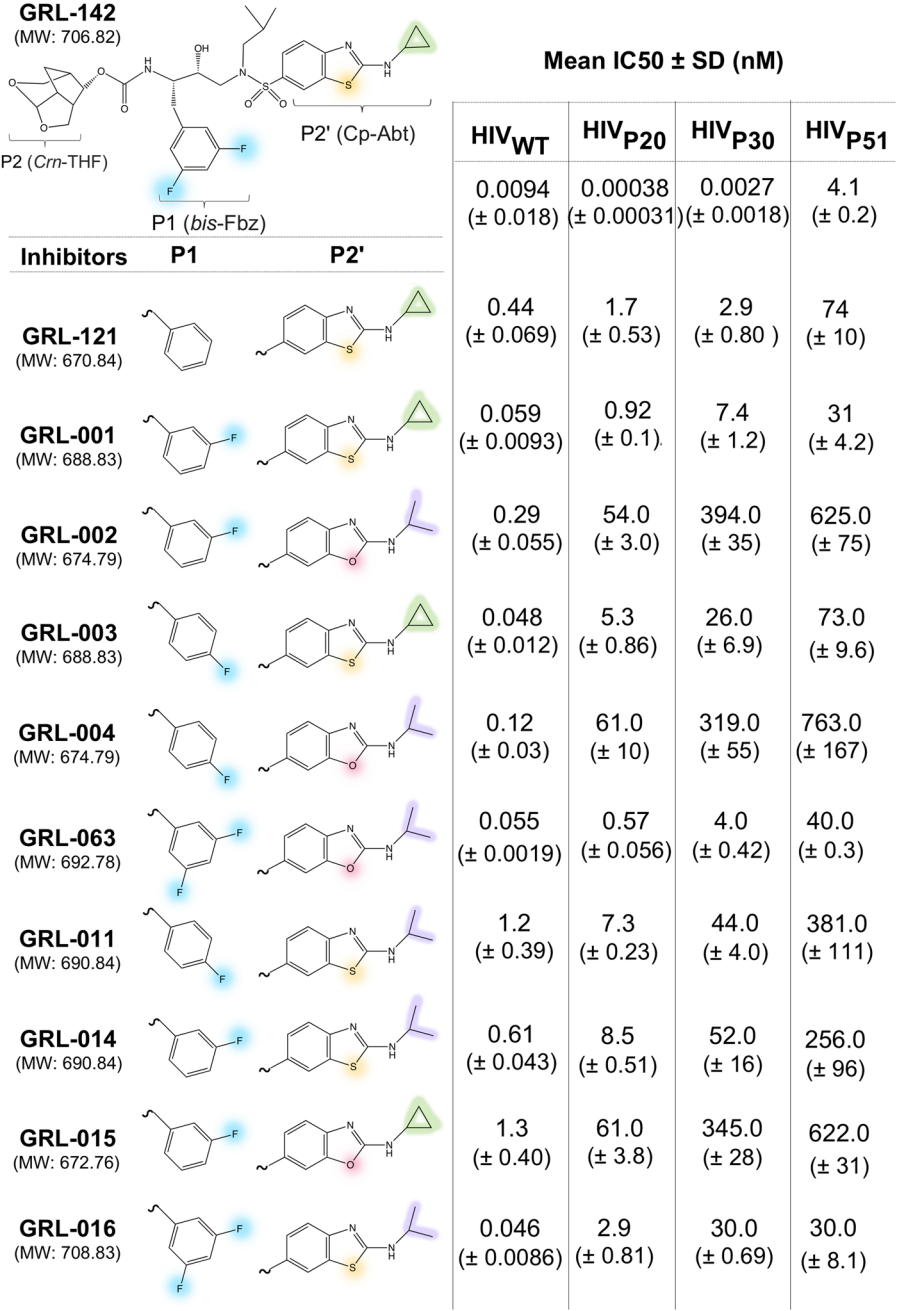
Chemical compositions of GRL-142 and its derivatives with their activity against HIV_WT_ and HIV_DRV_^R^_s_. The P2-*Crn*-THF moiety is present in all inhibitors. Modifications introduced at the P1 and P2′ positions are indicated with following colors; substitutions with fluorine atoms in P1 are highlighted in cyan, those of sulfur and oxygen in P2′ in yellow and salmon, respectively. Cyclopropyl and isopropyl moieties attached at the end of P2′ site are shown in green and purple, respectively.

Four recently reported PIs, designated GRL-142, GRL-121, GRL-001, and GRL-003 have been shown to occupy a larger surface in the binding pockets of PR, forming more extensive vdW contacts compared to darunavir^1,14^. In the present studies, we designed and synthesized seven novel PIs that incorporate the unique 6-5-5 ring-fused *crown*-like tetrahydropyranofuran (*Crn*-THF) as a P2-ligand as in GRL-142, GRL-121, GRL-001, and GRL-003. Further chemical variations were also introduced with a *meta*- or *para*-fluoro-substitution on the benzene ring at the P1 site and replacement of cyclopropyl-aminobenzothiazole (Cp-Abt) of GRL-142 with isopropyl-aminobenzothiazole (Ip-Abt) or isopropyl-aminobenzoxazole (Ip-Abo) as a P2′ ligand (Fig. 1).

## Results

**Figure 2:**
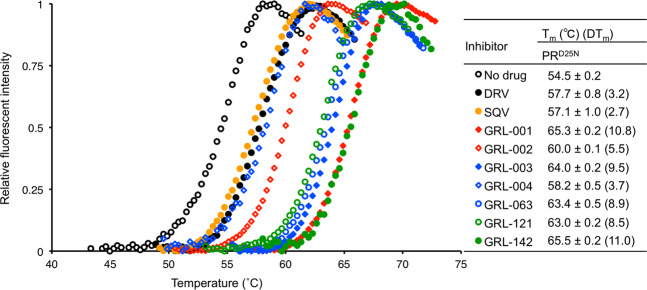
Thermal stability of PR^D25N^ in the absence and the presence of selected PIs. Note that the thermal stability curves with GRL-001, GRL-002, GRL-003, GRL-004, GRL-063, GRL-121, and GRL-142 significantly shifted toward higher temperatures (to the right) compared to those with no agent and those complexed with SQV or DRV.

We first determined anti-HIV-1 activity of GRL-142 and their derivatives against wild-type HIV_NL4-3_ (HIV_WT_) and three DRV-resistant HIV-1 variants (HIV_DRV_^R^) including HIV_DRV_^R^_P20_, HIV_DRV_^R^_P30_, and HIV_DRV_^R^_P51_. Amino acid substitutions in these variants associated with high-level resistance to multiple PIs including DRV are shown in Fig. S1. While early-stage amino acid substitutions (after 20 weeks of passage) occur mostly in distal region of PR, late-stage amino acid substitutions (after 30 and 51 weeks of passage) start to appear around the binding pocket. Overall, the eleven PIs examined in the present study exerted significantly greater anti-HIV-1 potency (up to ~10,000-fold)(Figs. 1 and S2 and Table S1) in cell-based assays and some of them including GRL-142, GRL-121, GRL-001 and GRL-003 proved to have much higher genetic barriers to the emergence of resistant variants as compared to DRV^1^. Although GRL-002 and GRL-004, both of which contain Ip-Abo, were less potent than GRL-142, GRL-121, GRL-001 and GRL-003, interestingly showed a high genetic barrier as well (Fig. S3),

Generally, the presence of two fluorine atoms in GRL-142 and its congeners (GRL-063 and GRL-016) appears to greatly contribute to their potent anti-HIV-1 activity against HIV_WT_ compared to the activity of GRL-121 that has no fluorine atoms. However, no definite difference was observed between the potency of the mono-*meta*-fluorine-containing inhibitor group (GRL-001, GRL-002, GRL-014, and GRL-015) and the mono-*para*-fluorine-containing inhibitor group (GRL-003, GRL-004, and GRL-011). Even though the potency of GRL-063 and GRL-016 against HIV-1 was progressively reduced from HIV_DRV_^R^_P20_ to HIV_DRV_^R^_P30_, and then to HIV_DRV_^R^_P51_, overall, these molecules exerted much better anti-HIV activity than mono-fluorine-containing inhibitors (Figs. 1 and 2). Of particular note, GRL-142 was remarkably potent against the three HIV_DRV_^R^_s_ and exerted even greater activity against HIV_DRV_^R^_P20_ and HIV_DRV_^R^_P30_ than against HIV_WT_ as previously reported^1^.

Structurally, the antiviral activity increased when the inhibitors had a cyclopropyl at the P2′-position as compared to an isopropyl together with an aminobenzothiazole moiety (see GRL-001 versus GRL-014 and GRL-003 versus GRL-011 in Figs. 1 and S2, and Table S1). Notably, substitution of P2′-cyclopropyl of GRL-142 with P2′-isopropyl, generating GRL-016, substantially reduced antiviral activity against HIV_WT_ by a factor of 5. Furthermore, the activity of GRL-016 was significantly weakened against HIV_DRV_^R^_s_ (Figs. 1 and S2, and Table S1).

We also determined the thermal stability of PR^D25N^ in the presence of DRV, saquinavir (SQV), GRL-001, GRL-002, GRL-003, GRL-004, GRL-063, GRL-121, and GRL-142 using differential scanning fluorimetry (DSF). As illustrated in Fig. 2, the melting point/denaturing temperature (T_m_) of PR^D25N^ alone was 54.5 °C; while in the presence of DRV and SQV, the T_m_ values increased to 57.7 °C and 57.1 °C, respectively. However, in the presence of GRL-001 through GRL-142, Tm values of PR^D25N^ dramatically increased, reaching temperatures ranging from 60.0 to 65.0 °C. These thermal stability results correlated well with the antiviral activity data, indicating that GRL-142 and its derivatives  bind much stronger to PR^D25N^ and stabilize the structure of PR^D25N^ at higher temperatures than DRV and SQV (Fig. 1).

**Figure 3:**
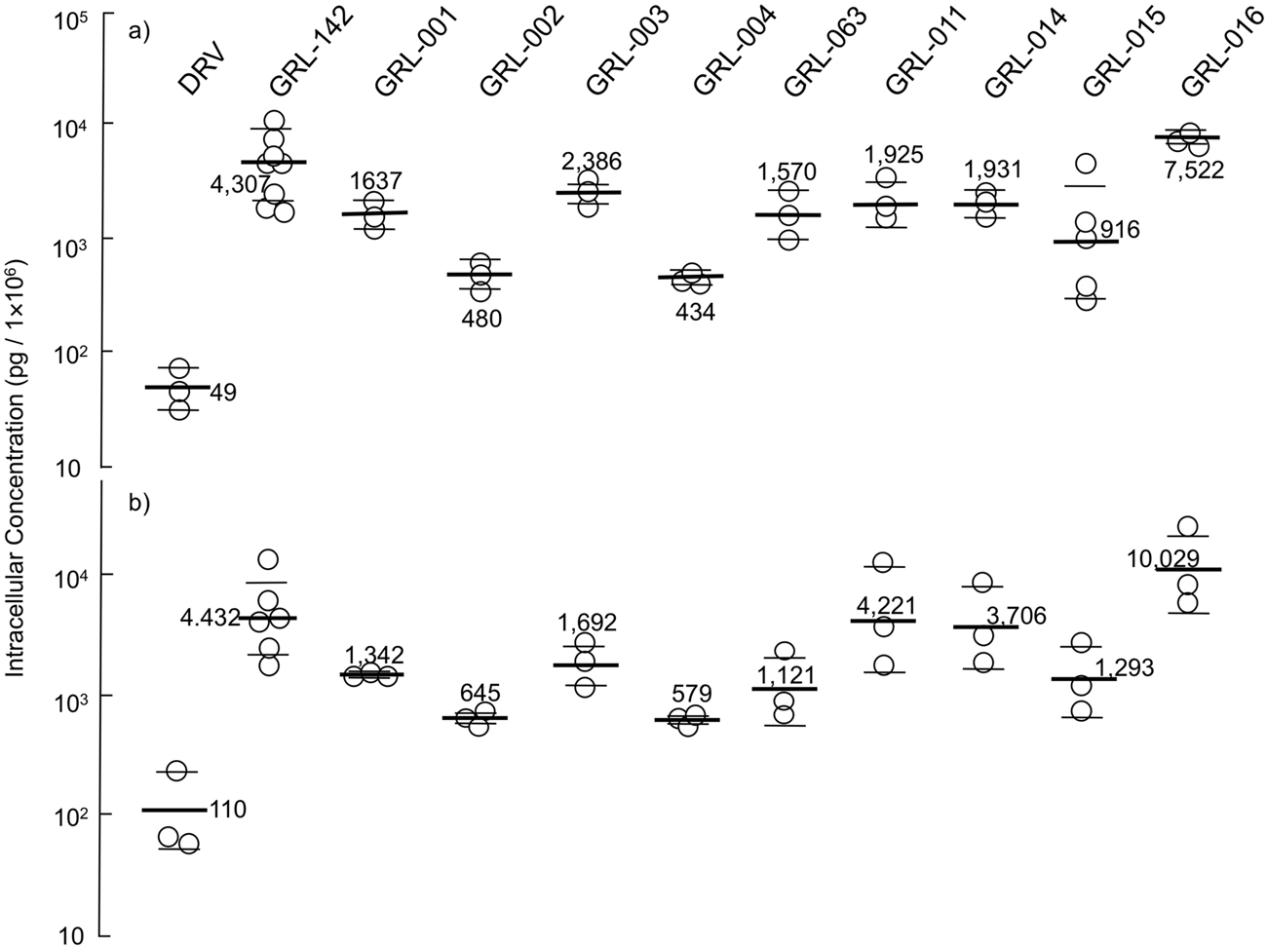
Cell membrane permeability of DRV, GRL-142 and nine GRL-142 derivatives. Cellular concentrations of each PI were determined using MT4 cells **(a)** and PBMC **(b)**. Thick bars denote geometric means of values from assays conducted on 3 to 8 different occasions, while short and thin bars denote ± 1 S.D. Each numeric value in the figure denotes the geometric mean value for each PI.

We further investigated the effects of fluorination and replacement of the P2′-Ip-Abo moiety on the cell membrane permeability (Fig. 3). The permeability across the cell membrane of PBMCs and MT4 cells for GRL-142 and the nine PIs was significantly higher than that of DRV. Especially, two *bis*-fluorine- and Abt-containing PIs, GRL-142 and GRL-016, showed 88- and 153-times greater cell permeability than DRV, followed by four mono-fluorine- and Abt-containing PIs (GRL-001, GRL-003, GRL-011, and GRL-014). No major difference in cell permeabilities was observed when the fluorine was at the *meta*- or *para*-position. All four Abo-containing PIs (GRL-002, GRL-004, GRL-063, and GRL-015) displayed reduced permeability compared to the six Abt-containing PIs mentioned above, independent of mono- or *bis*-fluorine moiety. Overall, GRL-016 that contains two fluorine atoms at the P1-benzene and P2′-Ip-Abt moiety, displayed the greatest cell permeability, suggesting that the combination of those two moieties is beneficial for permeability. In fact, GRL-011 with Ip-Abt had a 3.2-fold greater permeability than GRL-003 with Cp-Abt, and GRL-014 with Ip-Abt also had 3.4-fold greater permeability than GRL-001 with Cp-Abt, suggesting that Ip-Abt confers higher membrane permeability than Cp-Abt, at least among GRL-142 and its derivatives examined.

**Figure 4:**
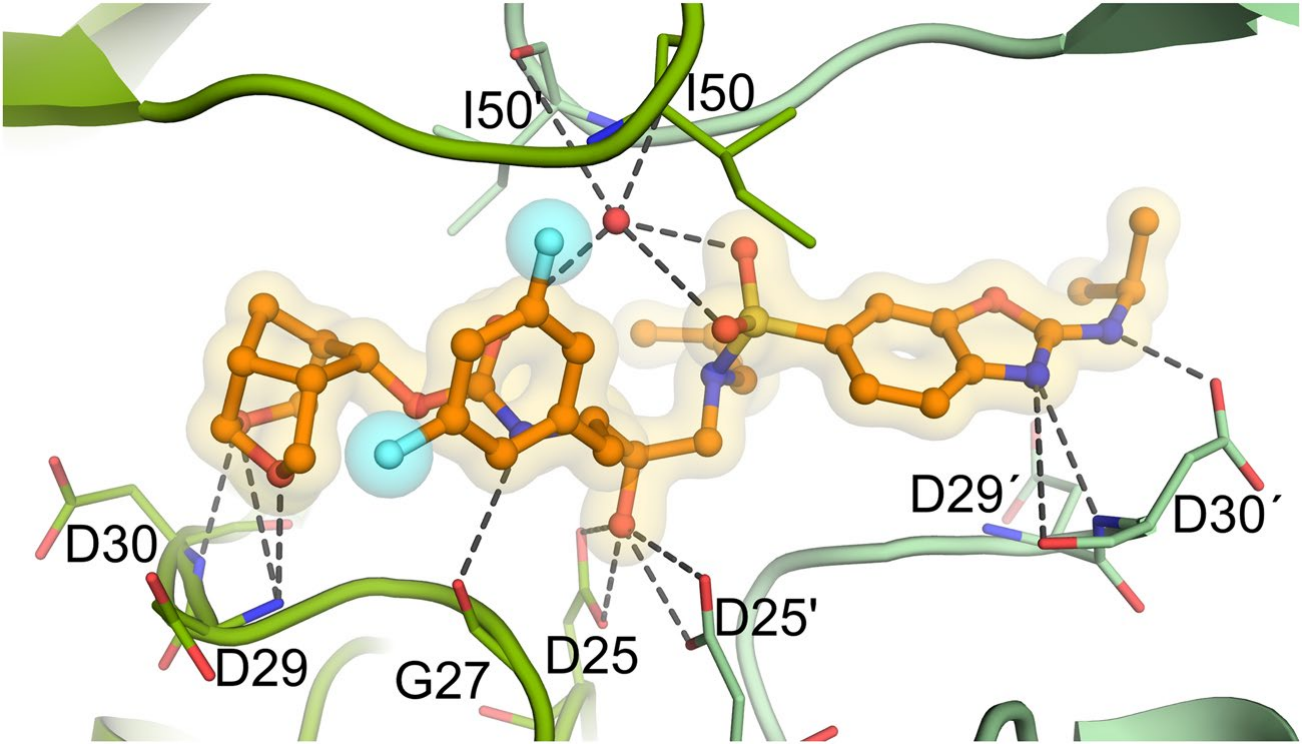
The X-ray crystal structure of GRL-063 bound to the active site of wild-type HIV-1 protease (PR_WT_). Carbon atoms of GRL-063 are shown in orange and subunits of PR_WT_ in green tones. Nitrogen, oxygen, sulfur, and fluorine atoms are shown in blue, red, yellow, and cyan, respectively. Hydrogen bond interactions that took place between GRL-063 and the protease residues are indicated by gray dashed lines, and two fluorine atoms are highlighted by cyan spheres.

We obtained structural insights into the intermolecular interactions between GRL-142 derivatives with PR, based on x-ray crystallography. Crystals of PR_WT_ complexed with three protease inhibitors (GRL-002, GRL-004, and GRL-063) as well as those of GRL-142^1^, GRL-001^14^ and GRL-003^14^ exhibited virtually the identical binding modes, which were found stabilized by a conserved hydrogen bond network (Fig. S4). The transition state hydroxyl group of all the PIs examined forms two hydrogen bonds with each of the catalytic aspartates, Asp25 and Asp25′; and on the opposite side, with the so-called “flap water” coordinated by the main-chain amide N-H groups of Ile50/Ile50′. The water molecule is present in all HIV-1 protease structures in complex with substrates or FDA-approved inhibitors except the one with tipranavir^15^. Tipranavir forms a direct hydrogen bond with the Ile50 residue and thereby contributes the stability of protease flap^16^. However, the number of direct hydrogen bond interactions between PR with the GRL-142 derivatives containing the *crn*-THF moiety discussed in the present work is greater than that with tipranavir (11 versus 9)^16^. The oxygen atoms of the *crn*-THF moiety form three direct hydrogen bonds to the main chain N-H of Asp29 and Asp30 residues in a similar manner as observed with the *bis*-THF moiety of DRV^7,17^. Fig. 4 and supplementary Video 1 show a representative feature of such interactions with GRL-063. Since the geometry and distance of *bis*-THF and *crn*-THF hydrogen bonds closely match, the better binding properties of *crn*-THF over *bis*-THF moiety are likely mediated by extensive vdW interactions with surrounding residues, such as Val32, Ile47, and Leu76 (Fig. S5). The *crn*-THF consists of two pentagonal rings and another hexagonal ring fits snugly inside the large cavity of the S2 site pocket; thereby establishing very effective vdW interactions in multiple directions (Fig. S5c). In fact, the importance of the THF group as a P2 site ligand was demonstrated with the early PIs containing various dipeptide isosteres^18,19^. Subsequently, a *mono*-THF group was incorporated into the prototypic PI, amprenavir^6^ (APV), but is too small to fully occupy the S2 sub-pocket and forms only one hydrogen bond to Asp29 (Fig. S5a), which was replaced with a bicyclic bis-THF ligand in DRV (Fig. S5b), and recently substituted to a stereochemically more complex form, the *crn*-THF in GRL-142 and 9 derivatives examined here (Fig. S5c).

**Figure 5:**
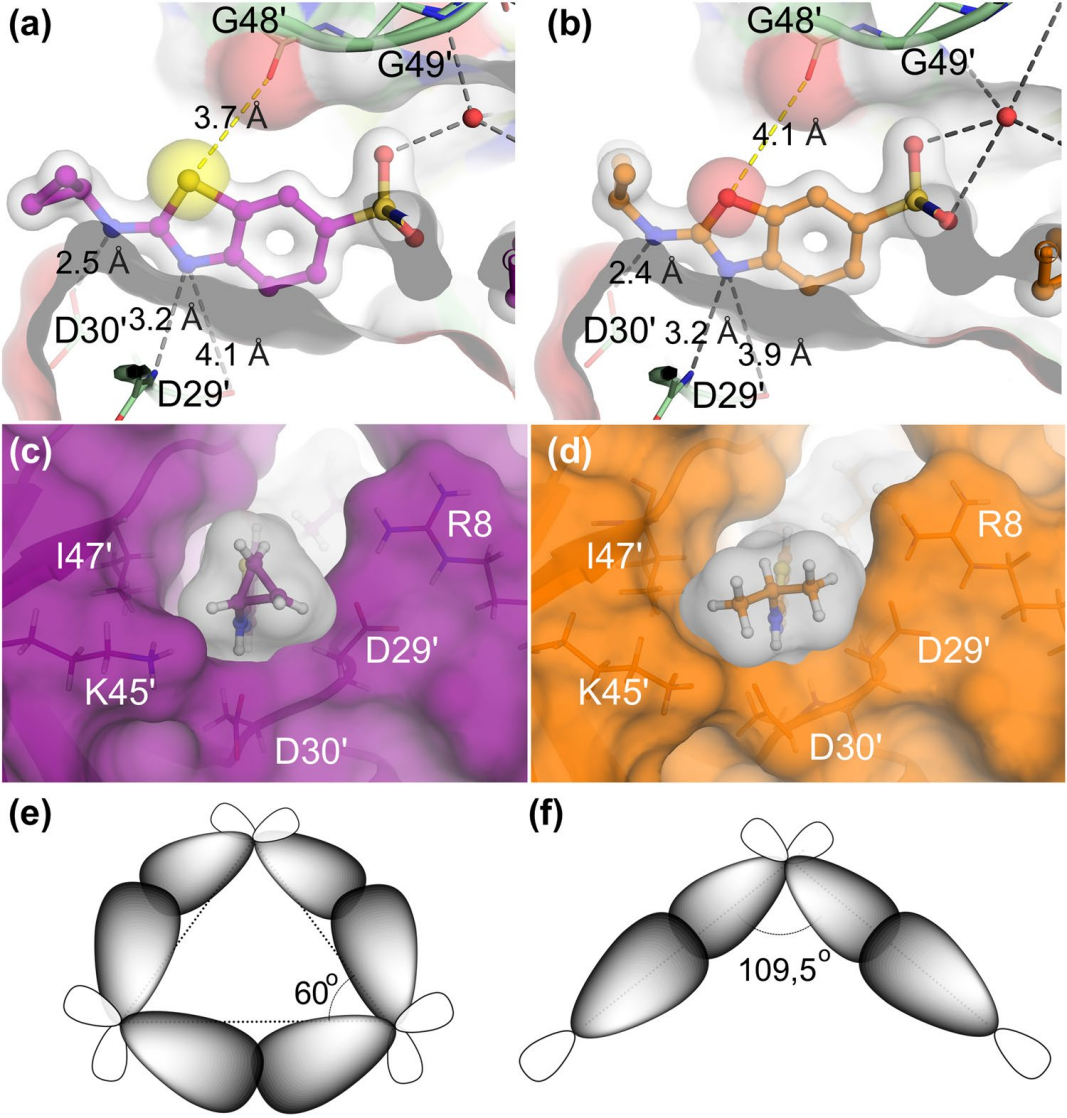
More favorable interactions are formed with the sulfur-atom-containing benzothiazole moiety over the oxygen-atom-containing benzoxazole moiety on the S2′ site. Structures of **(a)** benzothiazole moiety of GRL-142 and **(b)** benzoxazole moiety of GRL-063 inside the S2′ sub pocket of PR are shown. Hydrogen bonds are shown with gray dashed lines and the distances between sulfur atom (yellow) and oxygen atom (red) to the carboxyl oxygen of G48 are indicated with yellow dashed lines. Panel (c) shows the cyclopropyl moiety (magenta) of GRL-142 **(c)** and panel (d) shows the isopropyl moiety (orange) of GRL-063. Both groups are projecting out of the S2′ sub pocket of PR_WT_ and in contact essentially with the same residues. The isopropyl moiety is “larger” than cyclopropyl, and for this reason it pushes the flexible Lys45 side chain slightly away. Panel (e) shows a schematic representation of bent bonding that occurs within the chemical structure of cyclopropyl moiety, where the electron density accumulates in the center region due to the angle strain, which gives more room to maneuver to adapt to the binding pocket of PR_DRV_^R^_s_ than shown in **(f)**. By contrast, the isopropyl moiety forms more stable and inflexible state with typical alkane angle of 109.5^o^ as shown in panel (f).

In the S2′ subsite the inhibitor’s benzothiazole ring formed three hydrogen bonds with Asp-29′ and Asp-30′ of PR (Fig. 5a), thereby replacing less favorable indirect water-mediated interaction in was observed with aniline group of darunavir^20,21^. In addition, the sulfur atom of the benzothiazole ring forms a close contact with carboxyl oxygen of Gly-48 with a distance of 3.7 Å, producing significant interactions that should result in a coplanar arrangement of the amide and the thiazole ring (Fig. 5a). The sulfur atom is about twice as bulky than oxygen atom (atomic radius 100 pm for sulfur versus 60 pm for oxygen) and therefore the benzothiazole ring more efficiently occupies the binding pocket (Fig. 5a,b). The differences between GRL-001 and GRL-015 in the activity against HIV_WT_, HIV_DRV_^R^_P30_, and HIV_DRV_^R^_P51_ were approximately 22-, 47-, and 20-fold, respectively (Figs. 1 and S2, Table S1).

Of note, both isopropyl and cyclopropyl groups protrude at the edge of the S2′ site pocket where they heavily interact with the side chain of Lys45′ and to a lesser extent with Ile47′, Asp29′ and Asp30′ of PR (Fig. 5c,d). From a structural point of view the isopropyl moiety containing a wider C-C bond angle and two extra hydrogen atoms apparently fits well into the entrance of the binding pocket (Fig. 5d). However, the tight fit of isopropyl does not contribute anti-HIV-1 potency as much as cyclopropyl group (Figs. 1 and S2, Table S1). Obviously, the isopropyl renders the inhibitors bulkier, preventing their adaptation to conformational changes induced by amino acid substitutions in HIV_DRV_^R^_s_ structures (Fig. 5d). In contrast, the cyclopropyl moiety is more compact than isopropyl and by virtue of its rotational freedom due to the bent bonding, adapts better to the structural changes occurring in HIV_DRV_^R^_s_ structures (Fig. 5e,f)^22^.

**Figure 6:**
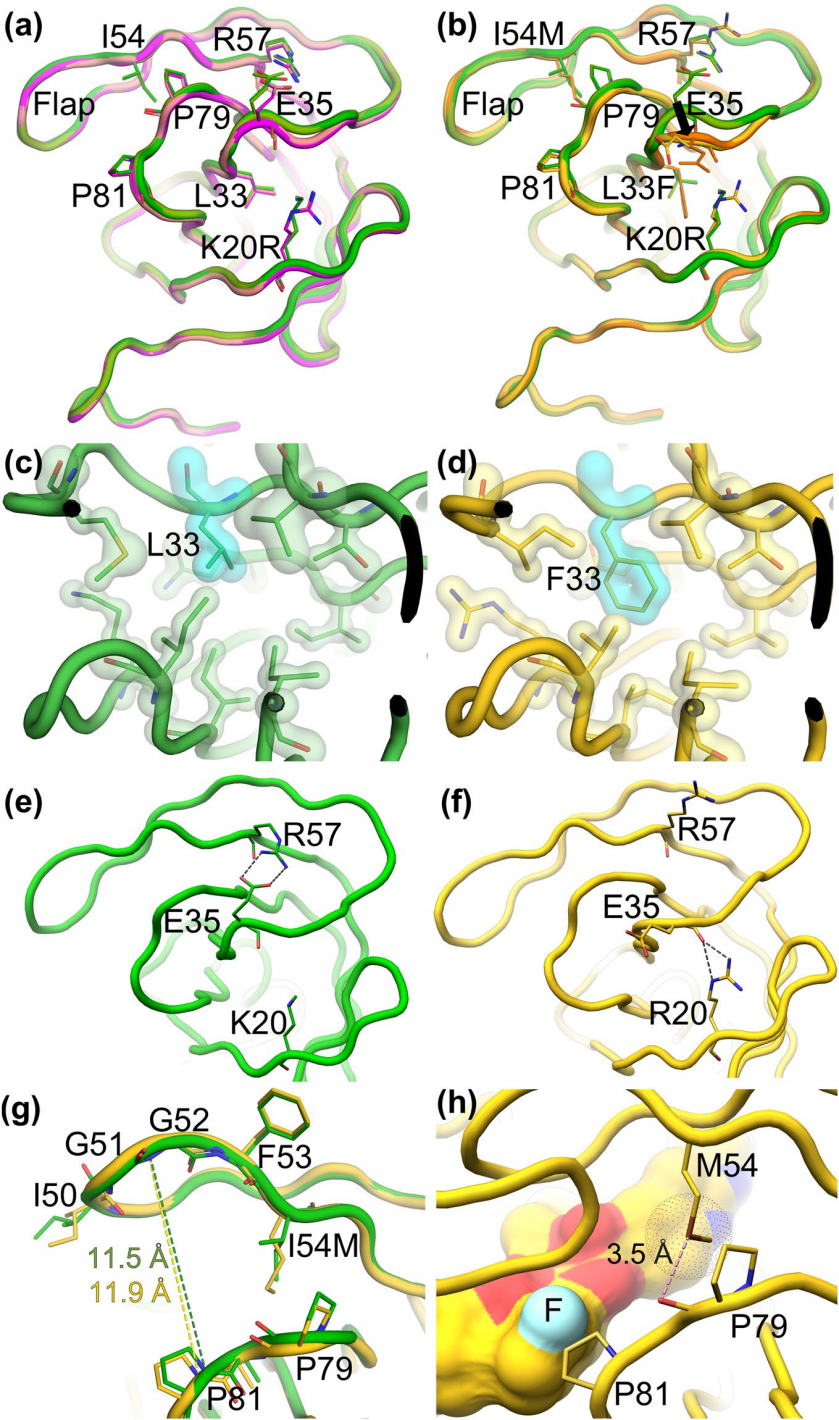
Structural deformations of PR revealed by overlaying of PR_wt_ with PR_DRV_^R^_P30_ or PR_DRV_^R^_P51_, Panel (a) shows a superimposition of two PR_WT_ structures in complex with GRL-001 or GRL-003 (light and dark green tones) onto two PR_DRV_^R^_P30_ structure in complex with GRL-001 or GRL-003 (magenta and salmon) exhibiting no major structural differences; while panel (b) shows superimposition of two PR_WT_ structures in complex with GRL-001 or GRL-003 (green tones) with two PR_DRV_^R^_P51_ structures in complex with GRL-001 and GRL-003 (yellow and orange), revealing significant structural differences around E35, as indicated by an arrow. Panels (c) and (d) focus on the hydrophobic cavity, where the substitution of L33 in PR_WT_ **[**(complexed with GRL-001 in green **(c)]** with the F33 in PR_DRV_^R^_P51_ **[**(complexed with GRL-001 in yellow **(d)]** causes the structural reorganization of the 30 s loop, resulting in a new hydrogen bonds pattern between E35 and R20 as shown with two grey dashed lines as shown in panel (f). Panel (e) shows the PR_WT_ structure in complex with GRL-001 (green), where the salt bridge between R57 and E35 connects the flap region on the top with the 30 s loops, stabilizing the flap region. However, in the PR_DRV_^R^_P51_ structures in complex with GRL-001 (yellow), two new hydrogen bonds are newly formed between E35 and R20, as described above, resulting in a loss of the salt bridge between R57 and E35 in the PR_WT_ structure. Panel (g), depicting a superimposition of PR_WT_ and PR_DRV_^R^_P51_, shows zoomed-in conformational changes around the flap region between PR_WT_ (green) and PR_DRV_^R^_P51_ (yellow) in complex with GRL-001. Distances measured for PR_WT_ (green) and PR_DRV_^R^_P51_ from amino group of P81 to amino group of G52 are indicated in green and yellow. Approximately 0.6 Å expansion of the binding pocket was measured. As shown in panel (h), depicting the structure of PR_DRV_^R^_P51_ complexed with GRL-001, the side chain interactions of M54 with particular emphasis to the sulfur-oxygen contact as well as sulfur-proline ring interaction are identified. GRL-001 is shown in surface representation with a fluorine atom highlighted in cyan directing towards P81, forming effective fluorine-based halogen bonds with P81 ring to counters the affinity loss caused by the amino acid substitutions in PR_DRV_^R^_P51_. (PDB IDs for GRL-001: 6MRC, GRL-003: 6MCS and GRL-142: 5TYS).

In order to understand the molecular mechanism of drug resistance, we compared the structure of PR_WT_ with mutant variants PR_DRV_^R^_P30_ and PR_DRV_^R^_P51_; each protein was crystalized in complex with an inhibitor, GRL-001 or GRL-003. X-ray structures of PR_WT_ complexed with GRL-001 and GRL-003 almost perfectly overlaid with each other (Fig. 6a,b). Similarly, the structures of DRV-resistance-associated protease variants, PR_DRV_^R^_P30_, overlaid very well with each other when complexed with GRL-001 and GRL-003 (Fig. 6a,b, respectively). Although 12 mutations occurred in the sequence of PR after 30 weeks passage, the PR_DRV_^R^_P30_ structures show no major structural deformation in comparison with PR_WT_. In the PR_DRV_^R^_P51_ structures, however, we observed significant structural differences around the 30 s loop (residues 29–35) (Fig. 6a,b). Actually, there are only three additional mutations present in the sequence of PR_DRV_^R^_P51_: L33F, I54M and A82I, strongly suggesting that the substitution L33F is a major trigger for these rearrangements around the 30 s loop. Interestingly, PR_DRV_^R^_P51_ structure shows high similarities around 30 s loop with previous reported structure of HIV-1 protease with L33F mutation (PDB code: 4YOB)^23^. The larger side chain of Phe33 forms stronger hydrophobic interactions inside the hydrophobic cavity that pulls the whole 30 s loop towards the segment around Arg20 (Fig. 6c,d). Consequently, the salt bridge between the residues of Arg-57 and Glu-35 in the PR_WT_ structures was lost in PR_DRV_^R^_P51_, and a new hydrogen bond between Glu-35 and Arg-20 was formed (Fig. 6e,f). Concomitantly, the 30 s loop lost its contacts with the flap region, which appears to become even more flexible, leading to a widened ligand binding pocket (Fig. 6g). This could be one of the main reasons for the reduced binding affinity of DRV towards PR_DRV_^R^_P51_, resulting in the emergence of DRV-resistance. Additional factor that contributes to the enlargement of the flap region is the substitution of the Ile-54 (in PR_WT_) with Met-54 (in PR_DRV_^R^_P51_). The large side chain of Met-54 slightly pushes the flap loop away from the 80 s loop (residues 79–84), causing a shift toward Pro-81 (Fig. 6g,h). These observations explain the importance of the *meta*- or *para*-positioned fluorine atom as in the case of GRL-001(Fig. 6h) and GRL-003^14^ that interacts with the side chain of Pro-81, probably compensating for the otherwise reduced activity of DRV towards HIV_DRV_^R^_P51_.

We summarize the contribution of each atom or moiety to the antiviral activity and cell permeability in Table 1. It shows that the combination of *bis*-fluorine, benzothiazole and cyclopropyl moieties all contribute to the greatest antiviral activity against HIV-1 strains.

**Table 1:** Schematic summary of contribution of each atom and moiety discussed in the present work.

DRV, GRL-142, and their analogs			Mean IC50 ± SD (nM)			
Inhibitors	P1	P2′	HIV_WT_	HIV_DRV_^R^_P20_	HIV_DRV_^R^_P30_	HIV_DRV_^R^_P51_
DRV (MW: 547.66)			5.1 (± 1.6)	42.0 (± 2.7)	342.0 (± 38)	>1000
GRL-142 (MW: 706.82)			0.0094 (± 0.018)	0.00038 (± 0.00031)	0.0027 (± 0.0018)	4.1 (± 0.2)
GRL-121 (MW: 670.84)			0.44 (± 0.069)	1.7 (± 0.53)	2.9 (± 0.80)	74.0 (± 10)
GRL-001 (MW: 688.83)			0.059 (± 0.0093)	0.92 (± 0.14)	7.4 (± 1.2)	31.0 (± 4.2)
GRL-002 (MW: 674.79)			0.29 (± 0.055)	54.0 (± 3.0)	394.0 (± 35)	625.0 (± 75)
GRL-003 (MW: 688.83)			0.048 (± 0.012)	5.3 (± 0.86)	26.0 (± 6.9)	73.0 (± 9.6)
GRL-004 (MW: 674.79)			0.12 (± 0.03)	61.0 (± 10)	319.0 (± 55)	763.0 (± 167)
GRL-063 (MW: 692.78)			0.055 (± 0.0019)	0.57 (± 0.056)	4.0 (± 0.42)	40.0 (± 0.3)
GRL-011 (MW: 690.84)			1.2 (± 0.39)	7.3 (± 0.23)	44.0 (± 4.0)	381.0 (± 111)
GRL-014 (MW: 690.84)			0.61 (± 0.043)	8.5 (± 0.51)	52.0 (± 16)	256.0 (± 96)
GRL-015 (MW: 672.76)			1.3 (± 0.40)	61.0 (± 3.8)	345.0 (± 28)	622.0 (± 31)
GRL-016 (MW: 708.83)			0.046 (± 0.0086)	2.9 (± 0.81)	30.0 (± 0.69)	30.0 (± 8.1)

*Meta*- or *para*-fluoro-substitution of fluorine almost equally contributed the cell permeability and antiviral activity, whereas the cell permeability and antiviral activity gained boost with dual fluorine at *meta* position. Benzothiazole-containing inhibitors performed overall better in their antiviral activity compared to benzoxazole-containing inhibitors. Cyclopropyl-containing inhibitors exerted greater antiviral activity than isopropyl-containing inhibitors.

## Discussion and Conclusions

The present investigation highlights intriguing structure-activity relationship, e.g., by replacement of a cyclopropyl with a saturated isopropyl moiety and/or the introduction of a fluorine atom(s), alters the potency against HIV-1. In particular, the present membrane penetration data confirm and strengthen our previous findings that the addition of two fluorine atoms greatly boosts the inhibitory properties of the PIs examined^1,14,24^. We have also examined to what extent the position of such fluorines affect their anti-HIV-1 activity. In addition to the effects described, dual fluorine substitutions may alter metabolic stability and cellular toxicity^25^, but these parameters were not examined in detail in the present study. The percentage of fluorinated therapeutics in the pharmaceutical market has markedly increased over the decades and in 2019, four out of ten approved new drugs contains at least one fluorine atom^26,27^. Among the nine FDA-approved PIs, however, only TPV contains three fluorines^4^. However, fluorine scan is increasingly being used for drug development^28^. To this end, our data may further contribute to the use of fluorine substitutions in the future design of novel PIs.

It was also noted in the current study that the inhibitors containing benzothiazole moiety at the P2′ position exerted greater anti-HIV-1 activity than those with benzoxazole moiety. This greater potency owes to the capacity of sulfur atoms forming bidirectional σ-hole potentials with the carbonyl oxygen of G48^29^. Sulfur is indeed one of the most prominent atoms in the chemical composition of FDA approved drugs^30^. Recent analyses of sulfur bonding interactions based on PDB structures has shown sulfur-based interactions most often take place with glycine backbone due to lack of steric hindrance^29^, which is apparently what we have seen with sulfur-Gly48 interaction.

Furthermore, the cyclopropyl-containing inhibitors exerted quite robust activity against most of the drug-resistant HIV-1 variant examined here. Replacement of cyclopropyl with isopropyl at the distal part of the inhibitor’s P2′ moiety results in a reduction in the anti-viral activity against wild-type HIV-1 (See GRL-001 versus GRL-014 in Fig. 1). In addition to well-recognized positive properties of cyclopropyl substitutions in drug design, such as increased metabolic stability and cell and blood-brain-barrier permeability^31^, our findings make an example of optimization approaches particularly against multi drug resistant targets. Comparison between cyclo- and iso-propyl groups in regard to cell membrane permeability remains to be elucidated; however, we observed a greater membrane permeability of compounds with an isopropyl group in the current study.

The majority of the FDA-approved PIs including DRV based on peptidomimetic structure^4^, our continuous efforts here resulted in a new set of inhibitors comprising more complex chemical arrangements derived from modifications on three moieties. Of note, the P2-*crn*-THF moiety occupies a larger three-dimensional space compared to DRV’s P2-*bis*-THF moiety (Fig. S2). The P2′-cyclopropyl moiety can rotate and therefore adapt to a better binding mode in the hydrophobic cavity of PR (Fig. 5c,d). Both *crn*-THF and benzothiazole moieties also snugly fill up the hydrophobic tunnel formed between HIV-1 protease dimer, resulting in extensive vdW interactions with HIV-1 protease compared to the case of DRV.

Although the X-ray-based structural differences between PR_WT_ and PR_DRV_^R^_s_ appear to be rather small and subtle, such differences are apparently sufficient to reduce the affinity of PIs to PR. By comparing the structures of PR_WT_ with PR_DRV_^R^_s_, we found various plausibly significant conformational changes which affect the binding pocket. Consequently, inhibitors have to adapt to the increased flexibilities of target proteins. Particularly, *bis*-meta fluorine and benzothiazole 2-amino cyclopropyl groups are indispensable components to sustain potent antiviral activity particulary against multi-drug resistant viruses. As a result of optimizing the P1, P2 and P2′ positions, the prototypic DRV has been converted to exceedingly potent PIs such as GRL-063 and GRL-016 as well as GRL-142^1^, GRL-001^14^, and GRL-003^14^. They appear to tolerate amino acid substitutions in the PR bindings pocket, making them promising PIs for future clinical development.

## Methods

### Synthesis - antiviral agents

Seven nonpeptidic PIs, GRL-002, GRL-004, GRL-063, GRL-011, GRL-014, GRL-015, and GRL-016, which contain mono-fluorine at the *meta* or *para* position in the P1-phenyl moiety or bis-fluorine in the P1-phenyl moiety, were newly synthesized. The method of synthesis of these protease inhibitors will be published elsewhere by A. K. Ghosh *et al*. The structural and biologic features of GRL-142^1^, GRL-121^1^, GRL-00^114^ and GRL-003^14^ were recently published by Aoki *et al*.^1^ and Hattori *et al*.^14^, respectively. Darunavir (DRV)^7,8^, lopinavir (LPV), and atazanavir (ATV) were purchased from Sigma-Aldrich.

### Cell culture and viruses

MT-4 cells were maintained RPMI 1640 medium containing 10% FCS, 50 U/ml penicillin and 50 µg/ml kanamycin. The following HIV-1 strains were used for the drug susceptibility assay and selection experiments shown as below as previously published^7,8,14^; HIV_NL4-3_, in laboratory-selected PI-resistant HIV-1 variants (_lab_HIV_PI_^R^s: HIV_SQV-5µM_, HIV_APV-5µM_, HIV_LPV-5µM_, HIV_IDV-5µM_, HIV_NFV-5µM_, HIV_ATV-5µM_, and HIV_TPV-15µM_), *in vitro*-selected DRV-resistant HIV-1 variants (HIV_DRV_^R^s: HIV_DRV_^R^_P20_, HIV_DRV_^R^_P30_ and HIV_DRV_^R^_P51_), and recombinant infectious clone derived from clinically isolated multi-drug-resistant HIV-1 variants (_rCL_HIVs: _rCL_HIV_F16_, _rCL_HIV_F39_, _rCL_HIV_V42_, _rCL_HIV_T45_, _rCL_HIV_T48_ and _rCL_HIV_F71_).

### Drug susceptibility assay

The susceptibility of HIV-1 to various drugs was determined as described previously^7,8,14^ with minor modifications. In brief, MT-4 cells (2 × 10^4^ cells/ml) were exposed to various HIV-1 strains (20 ng/mL of p24) and incubated for 7 days in the presence or absence of various concentrations of drugs in 10-fold serial dilutions. Assays were performed using Cell Counting Kit-8 (Dojindo, Japan) or lumipulse G1200 system with HIV-1 p24 antigen detection kit (Fujirebio, Japan) as following each manufacture’s instruction.

### Thermal shift assay

The thermal shift assay was conducted using protease variant PR^D25N^, which has no catalytic activity^14^. PR^D25N^ was refolded by adding 100 mM ammonium acetate stock solution (pH 6.0) to the final pH of 5.0 as previously published^14^. Then 0.005% Tween-20 was added to the refolded PR^D25N^ solution and concentrated to the 10 μM final concentration using Amicon Ultra-15 10 K centrifugal filter units (Millipore, Darmstadt, Germany). PR^D25N^ solution was supplemented with 50 µM of each tested compound. Final reaction mixture contained 100 mM ammonium acetate pH 5.0, 0.005% Tween-20, SYPRO Orange (5×) (Life Technologies) and 2.5% DMSO. Fluorescence intensity of each sample was scanned using the real-time PCR system 7500 (Applied Biosystems, Foster City, CA) starting from 15 up to 95 °C.

### *In vitro* selection of HIV-1 variants against GRL-002 and GRL-004

Selection of HIV-1 variants against GRL-002 and GRL-004 *in vitro* was conducted as previously published^14^. The wild-type HIV_NL4-3_ and a DRV-resistant HIV-1 variants were obtained after 30 *in vitro* passages in the presence of DRV (HIV_NL4-3_). These cells were propagated with increasing concentrations of each tested compound in MT-4 cells in a cell-free manner over 50 passages as follows: In each cycle, 1 ml of the cell-free supernatant containing viruses was harvested and transferred to 4 ml of culture medium containing fresh uninfected MT-4 cells in the presence of increased concentrations (1-, 2-, and 3-fold of previous cycle) of the drug for the next passage. Among those conditions in which the replication of HIV-1 in the culture was detected by substantial p24 Gag protein production (greater than 200 ng/ml increment), the highest concentrations were used to continue for the next round of culture. The emergence of highly drug-resistance was defined as >5 µM of drug concentration. Here the DRV, LPV, and ATV served as references.

### Determination of nucleotide sequences

Molecular cloning and determination of the nucleotide sequences of HIV-1 strains passaged in the presence of each compound were performed as previously described^1,14^ with slight modifications. DNA was isolated from HIV-1-infected MT-4 cells using the DNAzol DIRECT (Molecular Research Center, Cincinnati, OH) and used for amplification by PCR. Primers used for the first-round cover entire Gag-PR-encoding regions of the HIV-1 genome were LTR-F1 (5′-GAT GCT ACA TAT AAG CAG CTG C-3′) and PR12 (5′-CTC GTG ACA AAT TTC TAC TAA TGC-3′). The PCR mixture contained 10 μl Premix Taq (Premix Ex Taq Version2; Takara Bio Inc., Shiga, Japan), 1 μl proviral DNA solution, and 10 pmol of each PCR primer in a total volume of 20 μl. The products of the first round PCR were used for the second round of PCR. The second round PCR primers for the PR-encoding region were KAPA-1 (5′-GCAGGGCCCCTAGGAAAAAGGGCTGTTGG-3′) and Ksma2.1 (5′-CCATCCCGGGCTTTAATTTTACTGGTAC-3′). Final PCR products were purified using spin columns (MicroSpin S-400 HR columns; GE Healthcare Life Science., Pittsburgh, PA), and cloned directly into the pGEM-T easy vector (Promega) and subjected to sequencing using a model 3500 automated DNA sequencer (Applied Biosystems).

### Cellular drug uptake assays

PBMCs and MT4 cell extracts were prepared as previously described^32,33^. Briefly, each cell line (5 ×10^6^) was incubated at 37 °C for 60 min with each compound (10 µM final concentrations). Cells were harvested and washed 3 times with PBS. The final cell pellets were resuspended in 60% methanol and incubated for 5 min at 95 °C. Cell debris separated from solvent extract by spinning down at 13,000 rpm for 10 min and supernatant was evaporated overnight. The dried preparations were dissolved in DMSO (50 µL) and were analyzed for quantification of each compound using TOF LC/MS.

### TOF LC/MS analysis of PIs

Each sample (20 µl) was applied to Agilent TOF LC/MS and separated by VyDac C18 5-µm-particle-size column (3.2 mm by 150 mm). The column was previously equilibrated with 95% solvent A (water–0.01% formic acid) and 5% solvent B (acetonitrile 0.1% formic acid) with flow rate of 0.5 ml min-1. Following each injection, solvent B was increased by 2.5% per min to 55% over a 20 min period. Finally, solvent B was increased to 95% in 1 min and then returned to starting concentration over the next 1 min. The sodium adducts of each compound provided the most prominent peak and therefore were used for detection purposes whereas the parent ions provided the relative results. Compound obtained in the extracts was quantitated by comparing to molecular weight by mass spectrometry of each purified compound solved in DMSO as well as to elution profiles.

### Crystallization and Structure Determination

Purification and crystallization of HIV-1 protease were carried out as previously described^1,14^. X-ray data were collected at λ = 1.0 Å, 100 K, at SPring-8 (Hyōgo, Japan) and processed in HKL2000^34^ and DIALS^35^. The source wavelength for the data collection was 1.0 Å. Data collection statistics are shown in supplementary Table 2. The phase problem was solved by molecular replacement using phaser^36^ using the 2.0 Å structure of HIV-1 protease (PDB ID: 5TYS) as a model. All water molecules and ligand atoms were omitted from the starting model for the benefit of clarity. Subsequent cycles of refinements were performed in Refmac^37^ and PHENIX^38^. Coordinates and topology files of the inhibitors were generated using the Dundee PRODRG2 server^39^ and manually fitted to the electron density using Coot^40^. All structural figures were produced with PyMOL (version 1.3)^41^ and the Supplementary Video 1 was generated using UCSF Chimera^42^. The data collection and refinement details are provided in Supplementary Tables S2.

### Accession codes

The atomic coordinates and structure factors have been deposited into the Protein Data Bank with accession codes 6OYR, 6OYD, 6OGP, 6OGS, 6OGQ, 6OGT and 6OGL.

## Supplementary information

Supplementary Information.
