# Supplementary material for: Single atom changes in newly synthesized HIV protease inhibitors reveal structural basis for extreme affinity, high genetic barrier, and adaptation to the HIV protease plasticity
Source: Sci Rep. 2020 Jun 30;10:10664. doi: 10.1038/s41598-020-65993-z (PMC7326966; doi:10.1038/s41598-020-65993-z)
Supplement: Supplementary file 1 — Supplementary Information. [file 41598_2020_65993_MOESM1_ESM.pdf]

Single atom changes in newly synthesized HIV protease inhibitors reveal structural basis for extreme afffinity, high genetic barrier, and adaptation to the HIV protease plasticity

Haydar Bulut[a], Shin-ichiro Hattori[b], Hiromi Aoki-Ogata[a], Hironori Hayashi[b], Debananda Das[a], Manabu Aoki[a], David A. Davis[c], Kalapala V. Rao[d], Prasanth R. Nyalapatla[d], Arun K. Ghosh[d], and Hiroaki Mitsuya* [a,b,e]

[a] Experimental Retrovirology Section, HIV and AIDS Malignancy Branch, National Cancer Institute, National Institutes of Health, Bethesda, MD, United States.

[b] Department of Refractory Viral Infections, National Center for Global Health and Medicine Research Institute, Tokyo, Japan.

[c] Retroviral Disease Section, HIV and AIDS Malignancy Branch, National Cancer Institute, National Institutes of Health, Bethesda, United States.

[d] Department of Chemistry and Department of Medicinal Chemistry and Molecular Pharmacology, Purdue University, West Lafayette, IN, United States.

[e] Department of Clinical Sciences, Kumamoto University Hospital, Kumamoto, Japan.

ABSTRACT: HIV-1 protease inhibitors (PIs), such as darunavir (DRV), are the key component of antiretroviral therapy. However, HIV-1 often acquires resistance to PIs. Here, seven novel PIs were synthesized, by introducing single atom changes such as an exchange of a sulfur to an oxygen, scission of a single bond in P2'-cyclopropylaminobenzothiazole (or -oxazole), and/or P1-benzene ring with fluorine scan of mono- or bis-fluorine atoms around DRV's scaffold. X-ray structural analyses of the PIs complexed with wild-type PR (PR_{WT}) and highly-multi-PI-resistance-associated $PR_{DRV}{}^{R}{}_{P51}$ revealed that the PIs better adapt to structural plasticity in PR with resistance-associated amino acid substitutions by formation of optimal sulfur bond and adaptation of cyclopropyl ring in the S2'-subsite. Furthermore, these PIs displayed increased cell permeability and extreme anti-HIV-1 potency compared to DRV. Our work provides the basis for developing novel PIs with high potency against PI-resistant HIV-1 variants with a high genetic barrier.

Table of Contents

Figures and Tables	Page
Figure S1	3
Figure S2	4
Figure S3	5
Figure S4	6
Figure S5	7
Tables S1	8
Tables S3	9, 10
References	11

(a)

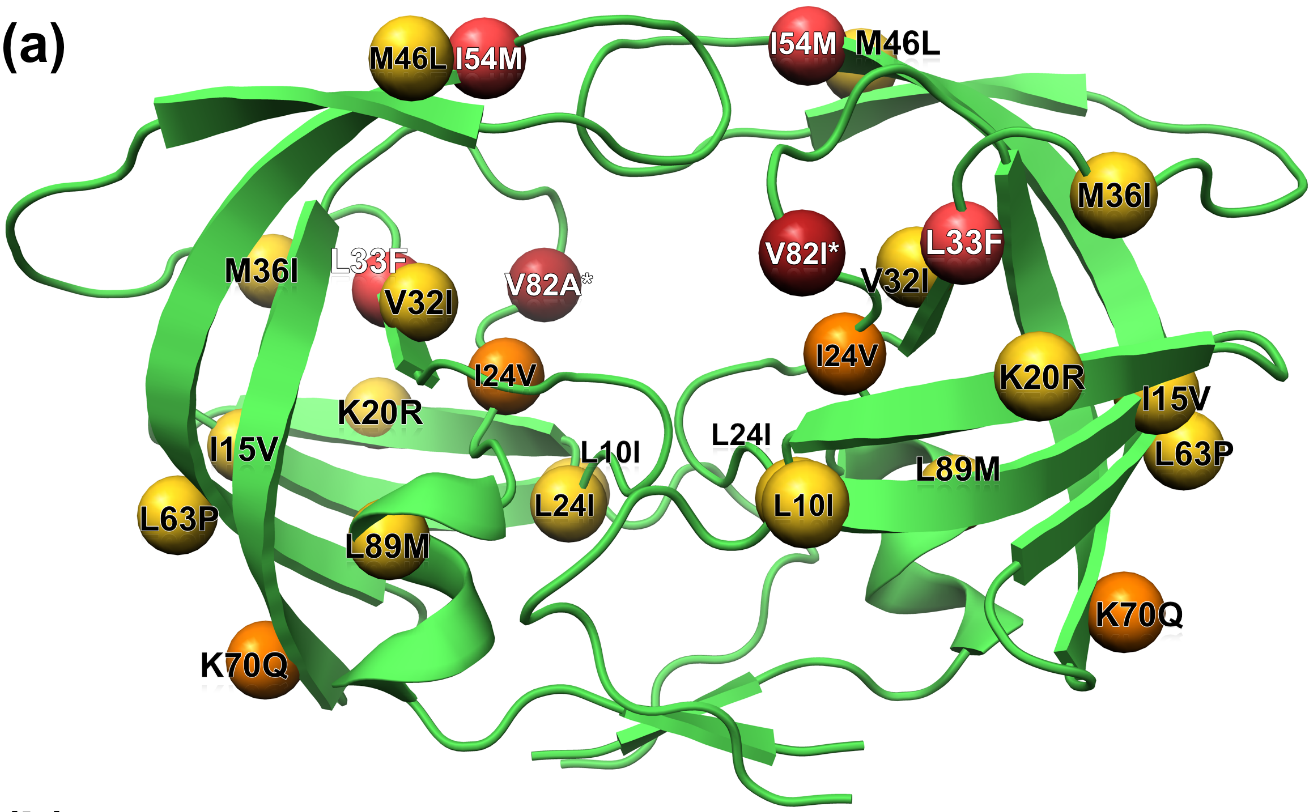

(b)

```
                        10         20         30         40         50
PR_WT          PQITLWQRPL VTIKIGGQLK EALLDTGADD TVLEEMNLPG RWKPKMIGGI
PR_DRV^R_P20   .........I ....V....R ...I...... .I...I.... .....L....
PR_DRV^R_P30   .........I ....V....R ...I...... .I...I.... .....L....
PR_DRV^R_P51   .........I ....V....R ...I...... .IF..I.... .....L....

                        60         70         80         90         99
PR_WT          GGFIKVRQYD QILIEICGHK AIGTVLVGPT PVNIIGRNLL TQIGCTLNF
PR_DRV^R_P20   .......... ..P....... .......... .A.....M.. .........
PR_DRV^R_P30   .......... ..P......Q .......... .A.V....M. .........
PR_DRV^R_P51   ...M...... ..P......Q .......... .I.V....M. .........
```

Figure S1: Amino acid substitutions associated with high-level resistance against multiple PIs including DRV. **(a)** Amino acid substitutions that appeared by 20 passages selection with DRV are shown by yellow spheres, substitutions that appeared after 30 passages selection are shown by orange spheres and those that appeared after 51 passages are shown by red spheres. The Val82 was first substituted to Ala by 20 passages, then was further substituted to Ile by 51 passages. **(b)** Two columns below show amino acid sequences of PR_{WT}, $PR_{DRV^R P20}$, $PR_{DRV^R P30}$, and $PR_{DRV^R P51}$.

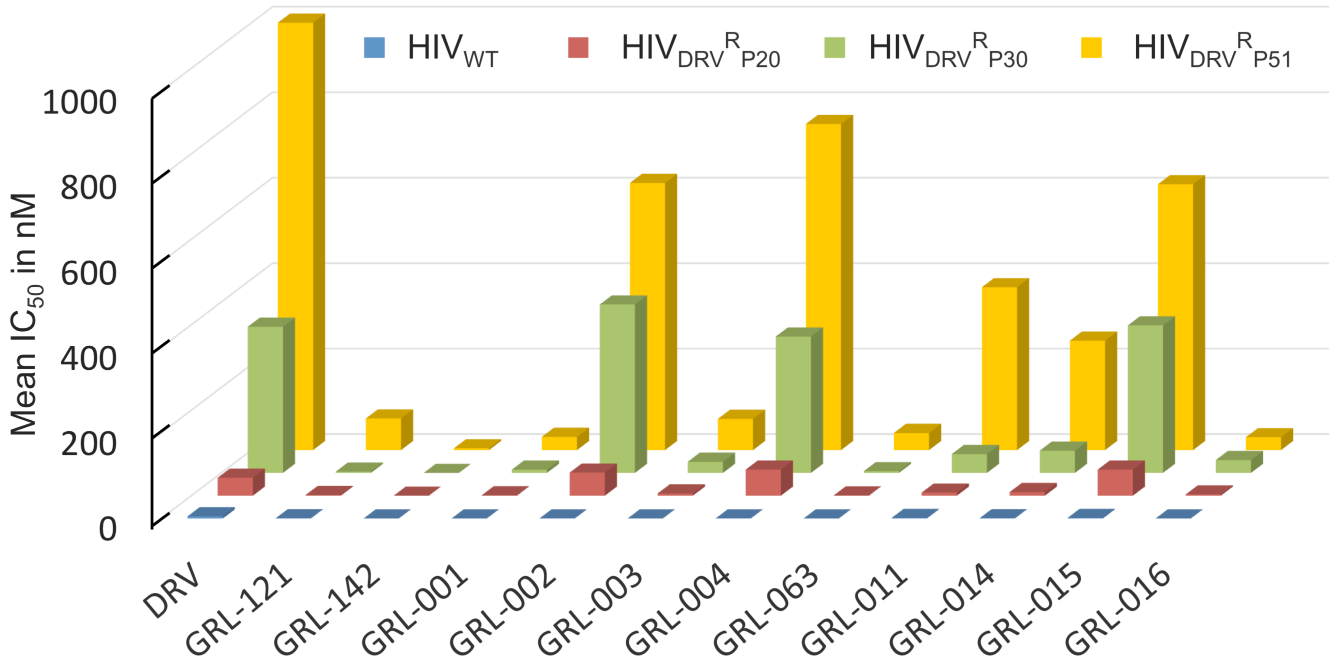

Figure S2. Antiviral activity of DRV, GRL-142, and nine GRL-142 analogs. Antiviral activity of each compound against HIV_{WT} and HIV-1 variants resistant to multiple PIs including DRV was determined using the levels of p24 production by target cells exposed to each virus and IC_{50} values of each PI was determined. See also Table 1 for exact IC_{50} values.

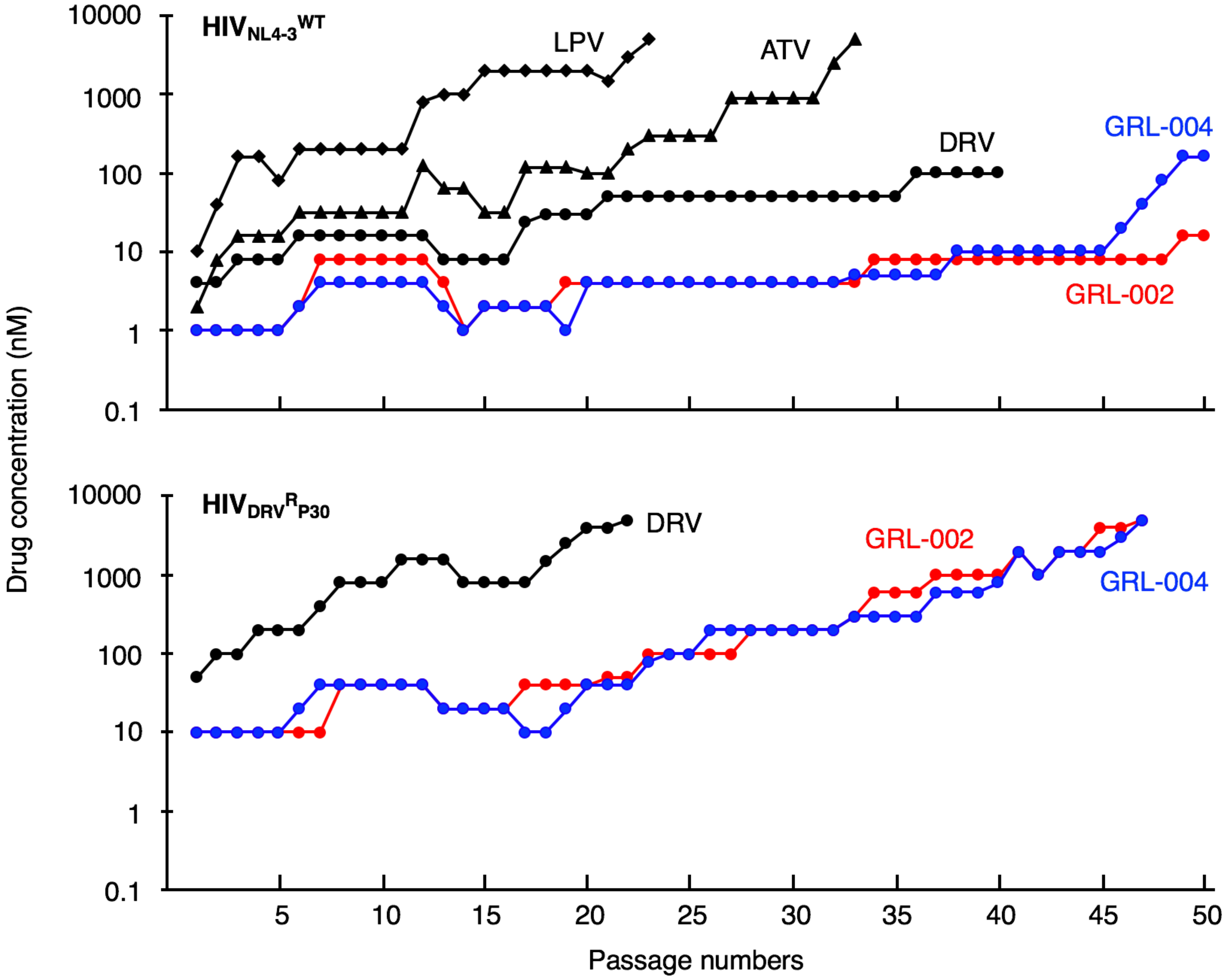

Figure S3. GRL-002 and GRL-004 have a high genetic barrier against the emergence of resistant HIV variants. The wild-type HIV_{NL4-3} (upper panel) and a DRV-resistant HIV-1 variant obtained from in vitro passage 30 with DRV ($HIV_{DRV}{}^{R}{}_{P30}$) (lower panel) were propagated in the presence of increasing concentrations of each compound in MT-4 cells in a cell-free manner over 50 passages[1]. Note that when HIV_{NL4-3} was selected with LPV and ATV, the virus started to propagate in the presence of high concentrations of those compounds. It is of note that when $HIV_{DRV}{}^{R}{}_{P30}$ was selected with DRV, the virus quickly started to propagate presumably through homologous recombination[2], but not with GRL-002 or GRL-004.

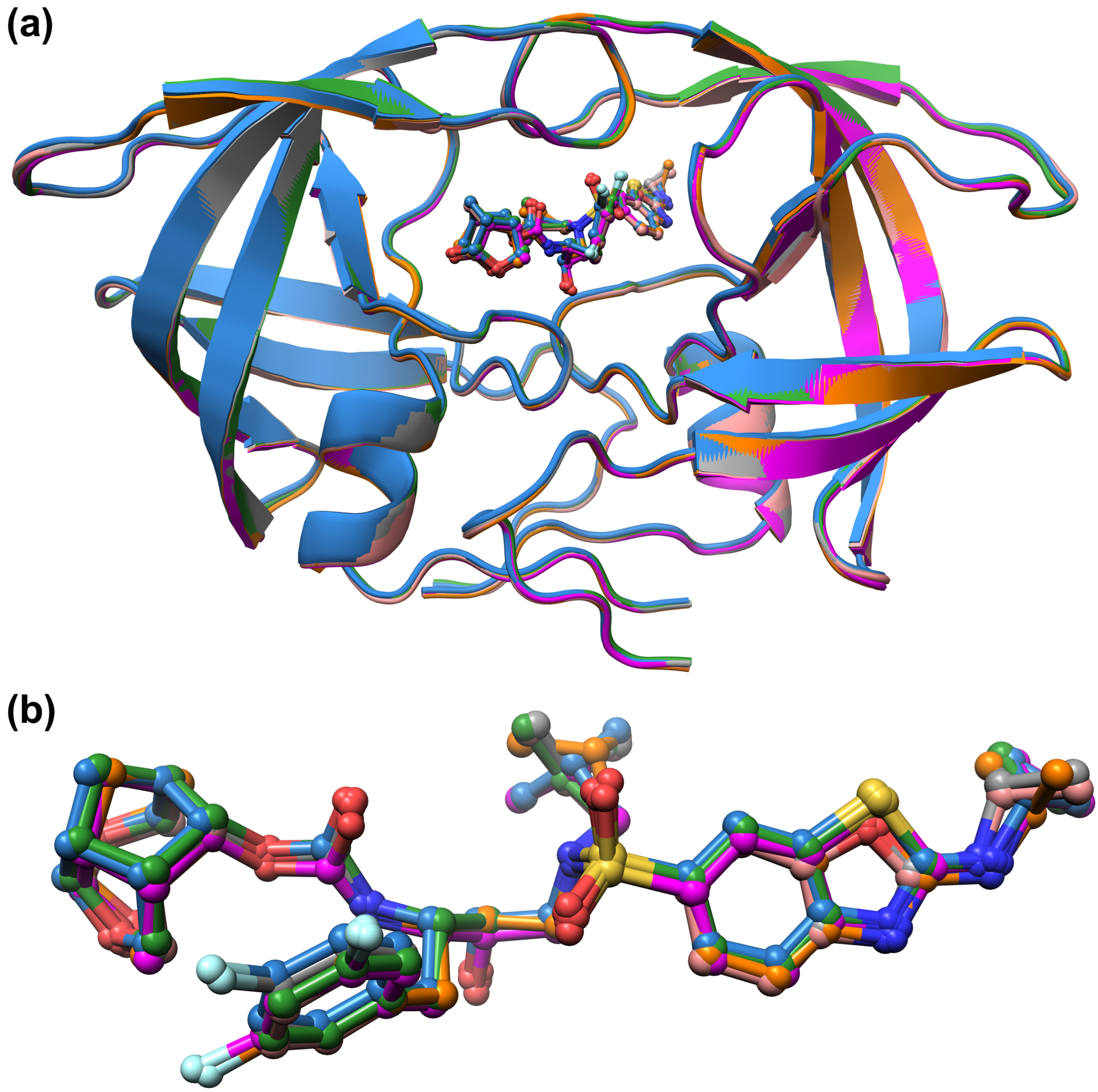

Figure S4. Structural profiles of GRL-142, GRL-001, GRL-002, GRL-003, GRL-004 and GRL-063 as complexed with PR_{WT}. (**a**) Superimposition of GRL-142 (magenta), GRL-001 (green), GRL-002 (salmon), GRL-003 (blue), GRL-002 (gray)and GRL-063 (orange) as complexed with PR_{WT}. **(b)** Zoomed-in picture shows overlay of inhibitors inside the binding pocket.

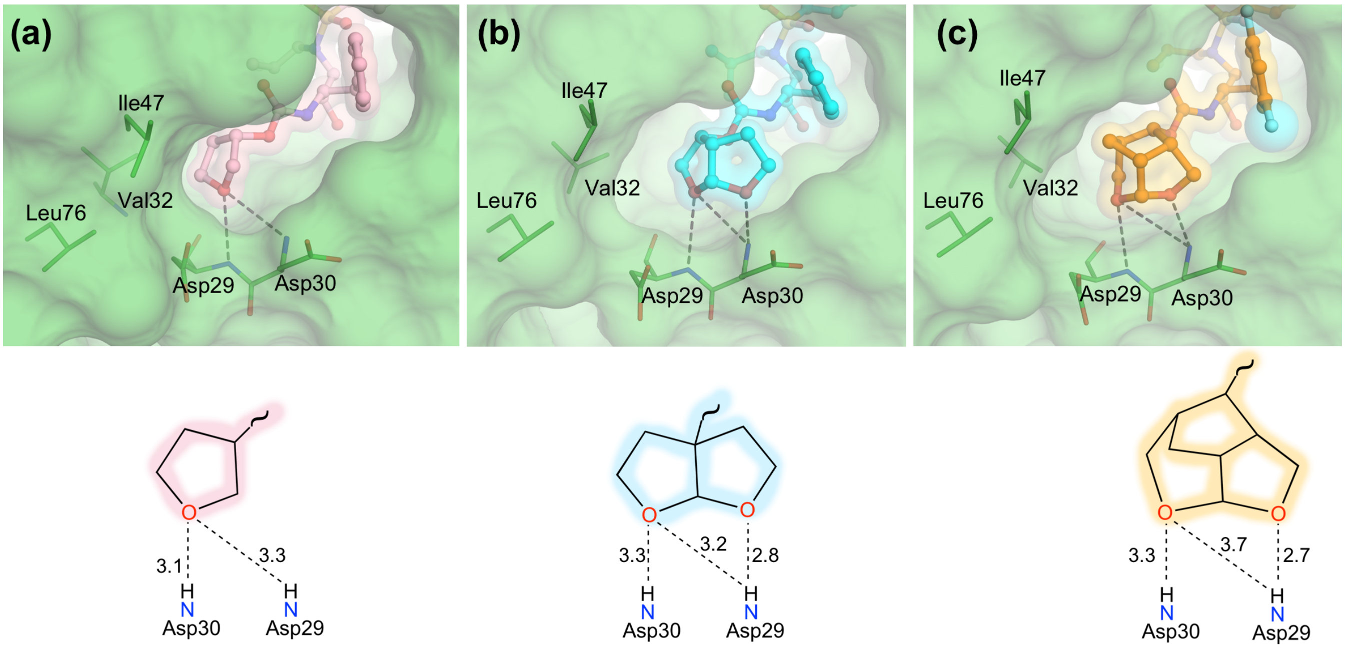

Figure S5. Comparison of **a)** *mono*-THF moiety of APV, **b)** *bis*-THF moiety of DRV and **c)** *crn*-THF moiety of GRL-063 inside the S2 sub pocket of HIV-1 protease. Lower panel shows a schematic depiction of hydrogen bond interactions and distances to the oxygens of each THF group, respectively. Although single oxygen containing *mono*-THF group is able to form two hydrogen bonds, two oxygen atoms containing *bis*-THF and *crn*-THF are able to form additional short hydrogen bond interactions with Asp29. It appears that the S2 sub pocket has some flexibility and slightly widens to accommodate the larger *crn*-THF group. (APV PDB ID: 3NU3, DRV PDB ID: 4HLA)

Table S1. Antiviral activity of DRV, GRL-142-13, and their analogs against HIV_{WT} and $HIV_{DRV}{}^{R}$ variants are shown. All assays were conducted in duplicate. The values shown denote the means ± 1 S.D.

	Mean IC50 ± SD (nM)			
**Inhibitors**	HIV_{WT}	$HIV_{DRV}{}^{R}{}_{P20}$	$HIV_{DRV}{}^{R}{}_{P30}$	$HIV_{DRV}{}^{R}{}_{P51}$
**DRV**	5.1 ± 1.6	42 ± 2.7	342 ± 38	>1000
**GRL-142**	0.017 ± 0.018	0.00041 ± 0.00031	0.0018 ± 0.0018	2.8 ± 0.2
**GRL-121**	0.44 ± 0.069	1.7 ± 0.53	2.9 ± 0.80	74 ± 10
**GRL-063**	0.055 ± 0.0019	0.57 ± 0.056	4.0 ± 0.42	40 ± 0.3
**GRL-001**	0.059 ± 0.0093	0.92 ± 0.14	7.4 ± 1.2	31 ± 4.2
**GRL-002**	0.29 ± 0.055	54 ± 3.0	394 ± 35	625 ± 75
**GRL-003**	0.048 ± 0.012	5.3 ± 0.86	26 ± 6.9	73 ± 9.6
**GRL-004**	0.12 ± 0.03	61 ± 10	319 ± 55	763 ± 167
**GRL-011**	1.2 ± 0.39	7.3 ± 0.23	44 ± 4.0	381 ± 111
**GRL-014**	0.61 ± 0.043	8.5 ± 0.51	52 ± 16	256 ± 96
**GRL-015**	1.3 ± 0.40	61 ± 3.8	345 ± 28	622 ± 31
**GRL-016**	0.046 ± 0.0086	2.9 ± 0.81	30 ± 0.69	30 ± 8.1

Table S2. Data collection and refinement statistics (molecular replacement)

Inhibitors_PRwt	**GRL-002_ PRWT**	**GRL-004_ PRWT**	**GRL-063_ PRWT**
PDB Entry	6OYR	6OYD	6OGP
**Data collection**			
Space group	P 61 2 2	P 61 2 2	P 61 2 2
Cell dimensions			
a, *b*, *c* (Å)	62.81, 62.81, 82.35	62.82, 62.82, 82.27	63.08, 63.08, 82.33
a, b, g (°)	90.00, 90.00, 120.00	90.00, 90.00, 120.00	90.00, 90.00, 120.00
Resolution (Å)	32.83 - 1.54 (1.595 - 1.54)*	54.41 - 1.46 (1.512 - 1.46)*	54.63 - 1.53 (1.585 - 1.53)*
Unique reflections	14722 (1419)	17208 (1660)	15202 (1483)
R_{sym} or R_{merge}	0.1034 (2.295)	0.1028 (2.698)	0.104 (2.767)
I / s*I*	15.72 (1.46)	14.13 (1.25)	15.37 (1.40)
Completeness (%)	99.32 (98.54)	99.63 (98.87)	99.94 (99.73)
Redundancy	18.6 (18.8)	18.6 (19.1)	18.5 (18.8)
**Refinement**			
Resolution (Å)	32.83 - 1.54	54.41 - 1.46	54.63 - 1.53
Reflections used in refinement	14710 (1416)	17203 (1657)	15199 (1483)
R_{work} / R_{free}	0.2224/ 0.2613	0.2257 / 0.2635	0.2285 / 0.2629
No. atoms	870	849	865
Protein	779	771	779
Ligand/ion	47	47	52
Water	44	31	34
B-factors	26.80	25.30	26.73
Protein	27.06	25.69	26.89
Ligand/ion	17.62	16.67	21.66
Water	31.92	28.67	30.65
R.m.s. deviations			
Bond lengths (Å)	0.023	0.017	0.019
Bond angles (°)	2.13	2.13	2.01

*Highest-resolution shell is shown in parentheses.

Inhibitor_ $PR_{DRV}{}^{R}{}_{P30}$	**GRL-001_ $PR_{DRV}{}^{R}{}_{P30}$**	**GRL-003_ $PR_{DRV}{}^{R}{}_{P30}$**	**GRL001_ $PR_{DRV}{}^{R}{}_{P51}$**	**GRL003_ $PR_{DRV}{}^{R}{}_{P51}$**
PDB Entry	6OGS	6OGQ	6OGT	6OGL
**Data collection**				
Space group	P 61 2 2	P 61 2 2	P 61 2 2	P 61 2 2
Cell dimensions				
a, *b*, *c* (Å)	62.93, 62.93, 81.67	62.82, 62.82, 82.15	63.07, 63.07, 82.19,	62.98, 62.98, 81.89
a, b, g (°)	90.00, 90.00, 120.00	90.00, 90.00, 120.00	90.00, 90.00, 120.00	90.00, 90.00, 120.00
Resolution (Å)	32.68 - 1.38 (1.429 - 1.38)*	32.78 - 1.41 (1.46 - 1.41)*	54.62 - 1.21 (1.253 - 1.21)*	45.4 - 1.21 (1.253 - 1.21)*
Unique reflections	25860 (2534)	19097 (1876)	28929 (2079)	29883 (2906)
R_{sym} or R_{merge}	0.132 (2.641)	0.1322 (2.796)	0.05492 (0.6966)	0.1687 (3.008)
I / s*I*	10.76 (1.22)	12.79 (1.10)	22.32 (0.89)	9.80 (1.13)
Completeness (%)	99.72 (99.24)	99.91 (99.73)	96.16 (70.97	99.81 (98.67)
Redundancy	18.4 (17.3)	18.2 (17.3)	14.3 (3.3)	17.2 (9.8)
**Refinement**				
Resolution (Å)	32.68 - 1.38	32.78 - 1.41	45.49 - 1.21	45.4 - 1.21
Reflections used in refinement	20223 (1968	19083 (1871)	28926 (2080)	29883 (2906)
R_{work} / R_{free}	0.2416 / 0.2796)	0.2118 / 0.2407	0.192 / 0.206	0.1989 / 0.2157
No. atoms	866	858	908	912
Protein	769	769	783	783
Ligand/ion	47	47	57	57
Water	50	42	68	72
B-factors	23.52	19.84	17.46	16.86
Protein	23.46	19.95	16.82	16.14
Ligand/ion	15.14	12.61	15.54	15.22
Water	32.42	25.91	26.39	25.90
R.m.s. deviations				
Bond lengths (Å)	0.017	0.017	0.016	0.018
Bond angles (°)	2.21	2.05	1.91	2.11

*Highest-resolution shell is shown in parentheses.

REFERENCES

1. Hattori, S. I.; Hayashi, H.; Bulut, H.; Rao. K. V.; Nyalapatla. P. R.; Hasegawa. K.; Aoki, M.; Ghosh, A. K.; Mitsuya. H., Halogen Bond Interac-tions of Novel HIV-1 Protease Inhibitors (PI) (GRL-001-15 and GRL-003-15) with the Flap of Protease Are Critical for Their Potent Activity against Wild-Type HIV-1 and Multi-PI-Resistant Variants. Antimicrob Agents Chemother. **2019**, 24;63(6)
2. Aoki, M.; Hayashi, H.; Rao, K. V.; Das, D.; Higashi-Kuwata, N.; Bulut, H.; Aoki-Ogata, H.; Takamatsu, Y.; Yedidi, R. S.; Davis, D. A.; Hatto-ri, S. I.; Nishida, N.; Hasegawa, K.; Takamune, N.; Nyalapatla, P. R.; Osswald, H. L.; Jono, H.; Saito, H.; Yarchoan, R.; Misumi, S.; Ghosh, A. K.; Mitsuya, H., A novel central nervous system-penetrating protease inhibitor overcomes human immunodeficiency virus 1 resistance with unprecedented aM to pM potency. Elife. **2017**, 6.
